# Comparing Advanced with Basic Telerehabilitation Technologies for Patients with Rett Syndrome—A Pilot Study on Behavioral Parameters

**DOI:** 10.3390/ijerph19010507

**Published:** 2022-01-03

**Authors:** Rosa Angela Fabio, Martina Semino, Samantha Giannatiempo, Tindara Caprì, Giancarlo Iannizzotto, Andrea Nucita

**Affiliations:** 1 Clinical and Experimental Medicine Department, University of Messina, 98121 Messina, Italy; rosaangela.fabio@unime.it (R.A.F.); tindara.capri@unime.it (T.C.); 2 Airett Innovation and Research Center (CARI), 37122 Verona, Italy; martina.semino@airett.it (M.S.); samantha.giannatiempo@gmail.com (S.G.); 3 Institute for Biomedical Research and Innovation (IRIB), National Research Council of Italy (CNR), 98164 Messina, Italy; 4 LuxAI S.A., 1724 Luxembourg, Luxembourg; 5 Department of Cognitive Science, Psychology, Education and Cultural Studies, University of Messina, 98122 Messina, Italy; giancarlo.iannizzotto@unime.it

**Keywords:** Rett syndrome, telerehabilitation, telemedicine, multiple disabilities, human-computer interaction

## Abstract

The aim of this study is to compare the performances of patients with Rett syndrome that were undergoing advanced telerehabilitation (ATR) and patients that were undergoing basic telerehabilitation (BTR). It was hypothesized that patients that were undergoing ATR training would have better improvement in nearly all the motor and cognitive scale scoring activities that were administered, thus showing reduced disability. A total of 20 young girls and women with a diagnosis of RTT, ranging from age 4 to 31 years old (Median: 12.50; IQR: 9.50–17.25) underwent a pre-test, treatment post-test 1, treatment, and post-test 2 procedure. The treatment consisted of either ATR or BTR, lasting 10 weeks with three sessions a week of about an hour. The results showed that the group with advanced telerehabilitation improved their performance better than the control group only in some neuropsychological measurements. The results are discussed in the light of critical factors of telerehabilitation.

## 1. Introduction

Telerehabilitation (TR) is the delivery of rehabilitation and therapeutic services with the support of various technologies, such as videos, websites, computer programs, and videoconferencing platforms [1]. TR also refers to the use of information and communication technologies (ICT) to provide rehabilitation services remotely to people at home or in other remote locations [2]. Such services include therapeutic interventions, remote monitoring of progress, education, consultation, and training [3].

The rapid evolution of technology has allowed health professionals to provide healthcare services in new and different applications. Consequently, different technologies are used in the TR interventions: from common, basic tools (i.e., phone, videoconference) to more advanced devices (i.e. wearable sensors, 3D camera, eye tracker, and virtual reality) [4]. Essentially, TR applications can be divided into two main strands: basic applications include telephone- or videoconference-based assessment, treatment, and management services; advanced applications include two-way real-time interaction visits with live audio and video, asynchronous e-visits, and virtual reality [4].

The type of technology that is used in TR interventions can both negatively and positively influence the effectiveness of TR; for example, basic telerehabilitation technology, such as a video conferencing system, has been found difficult to use for people who typically do not use this type of technology [5,6]. In contrast, some studies have demonstrated that advanced technologies together with TR applications can obtain better results [7,8].

TR has been used in different clinical fields [9,10]. Concerning the field of neurodevelopmental disorders (NDDs), a recent systematic review has identified three categories of technology that are used in TR interventions for children with NDDs: integrated systems, games-based technologies, and video-based technologies [11]. This review has demonstrated that TR is an effective tool in improving the adaptive skills of children with NDDs. Moreover, literature show that TR can be a promising intervention for children with neurological diseases [12] as well as for the treatment of motor [13,14], cognitive [15], and language disorders [3,16,17,18,19,20,21]. Another recent review [22] has shown evidence of the efficacy of providing TR interventions to parents and children with NDDs. It has also highlighted that it is necessary to understand what is suitable for clinical adoption among the different technologies. Another review reported that the benefits of advanced TR include more real-life support for rehabilitation, major reduction of burden on parents, continuous feedback to the child, and greater self-control of competence [23]. Moreover, it was found that the use of advanced TR is a promising approach for improving cognitive, motor, and linguistic abilities during the same session of treatment [23]. This is an important factor given that children with NDDs can show multiple disabilities.

Taken together, the review studies on advanced TR evidenced the affordability, effectiveness, and suitability of the use of advanced technologies to improve cognitive, emotional, motor, and adaptive skills of children and adolescents with NDD [24]. These promising results represent a solid theoretical background for the use of advanced technologies as unprecedented opportunities for supporting the implementation of TR services and the development of best practices for patients with NDDs. However, it is important to note that the application of both advanced and basic TR technologies to patients with cerebral palsy and rare genetic diseases (e.g., Rett Syndrome) has not been sufficiently investigated [24].

In particular, although the use of TR for NDDs has grown rapidly, few studies use TR intervention for children with Rett Syndrome (RTT) [20,25,26]. Probably, this can be related to the fact that RTT is a rare genetic disorder and it is also a heterogeneous syndrome with different levels of severity, including cognitive, language, motor deficits, neurological, and behavioral impairments [27,28]. Moreover, patients with RTT show deficits in attention, so the educator or therapist has to provide intervention to stimulate the attention of these patients and reduce stereotypies [29,30]. Given the complexity of RTT, treatments require a multidisciplinary team with specialists coming from different areas: medical, psychological, social, educational, and occupational, and are directed toward symptoms and providing support in different areas of daily life. Consequently, it can be difficult to develop a global intervention using TR that is aimed to treat different skills.

According to previous results in NDDs [11,22,24], the use of TR also appears to be suitable for patients with RTT. The main aim of the present study is to determine whether the use of TR with advanced technology (ATR) in patients with RTT leads to greater (or equal) improvements in motor and cognitive functions than basic TR (BTR).

The underlying logic is that the ATR system is equipped with support for eye-tracking so that both the therapist and caregiver can monitor the choices of the patient with high precision and rely on the support of video recordings and a 3D reconstruction in real-time that are produced by applying computer vision and artificial intelligence (AI) techniques. The therapist and the caregiver can also see the reconstructed patient’s skeleton superimposed onto the patient image in the video; together, these enrichments allow greater involvement on the part of therapists, caregivers, and patients.

To achieve this aim, a cohort of patients with RTT were assigned to two groups that were matched for levels of severity and functional abilities: patients that were undergoing advanced telerehabilitation (ATR) and patients that were undergoing basic telerehabilitation (BTR). It was hypothesized that the patients that were undergoing ATR training would have greater improvement in nearly all the motor and cognitive scale scoring activities that were administered, with reduced disability. More in-depth, we expected that: (1) the level of attention of the ATR group would increase with concomitant decreases of stereotypies and trainer interventions; (2) the basic behaviors-prerequisites area, neuropsychological area, basic cognitive area, advanced cognitive area, communication area, emotional area, hand motor area, graphomotor area, global motor area, and autonomy in daily life area, would all increase; (3) these improvements would have an impact also on the general scales that measure the level of severity of the disease and the level of functional abilities.

## 2. Materials and Methods

### 2.1. Participants

A total of 20 young girls and women with a diagnosis of RTT, ranging from age 4 to 31 years old (Median: 12.50; IQR: 9.50–17.25) were recruited from the Italian Rett Association (AIRETT). All the participants were born into non-consanguineous marriages. Regular immunizations had been carried out. At birth, the weight and height were all normal. The RTT patients were classified as clinical stage III (characterized by prominent hand apraxia/dyspraxia, apparently preserved ambulation ability, and some communicative ability, mainly eye contact) or stage IV (late motor deterioration, with progressive loss of ambulation ability), according to the criteria for classic RTT by Hagberg et al. [31]. All the participants showed pervasive hand stereotypies. All attended schools or socio-educational centers. A general assessment was carried out by a psychologist using the Vineland Adaptive Behavior Scale (VABS) [32] and the Rett Assessment Rating Scale (RARS) [33,34,35,36]. Table 1 shows the characteristics of the groups. 

The Mecp2 mutation was seen in most of the participants; specific mutations of the Mecp2 gene were: 40% showed T158M, 20% showed P322L, 15% showed R255X, and 15% showed P152R; for 10%, it was not possible to specify the type of Mecp2 gene mutation. With reference to the eligibility criteria of the participants from the Rett population, inclusion criteria of the participants with Rett syndrome, are that they had to be able to stay seated to independently watch the presented stimuli or with some support. The exclusion criteria refer to patients with genetic mutations FOXG1 and CDKL5.

The participants were matched for age, severity level of the disease, and functional ability level and randomly assigned to the ATR and BTR rehabilitation groups.

We asked the reference neuropsychiatry of each patient to give a medical judgment of severity based on the typical characteristics of the syndrome (epilepsy, mood swings, convulsions, aerophagia, scoliosis). The severity level ranged from 5 (mild severity) to 20 (severe severity). The mean severity index about the typical characteristics of the syndrome was 9.

### 2.2. Study Design

**Figure 1:**
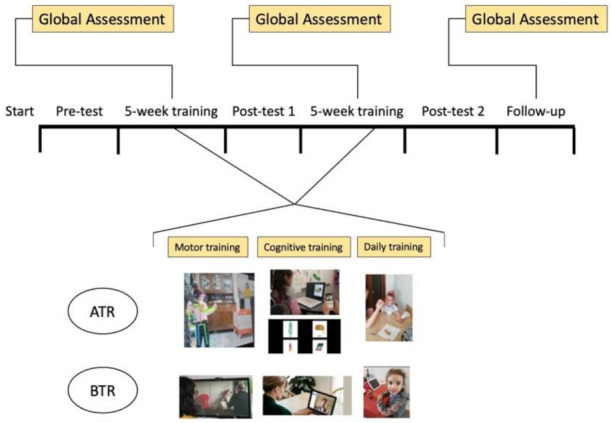
The study design with all phases. In ATR, the monitoring of the skeleton and eye-tracking are shown, while in the BTR group, only the video-call is shown.

This study employed a pre-test, post-test 1, and post-test 2 design with two groups: the control group and the experimental group (Figure 1). 

The former received evaluation and treatment with the use of BTR (a simple online Skype platform), whereas the latter received evaluation and treatment with the use of an ATR system that was equipped with enhanced tools to acquire eye-gaze data and a reconstructed patient’s skeleton that was superimposed on the patient image in the video. In the pre-test phase, all the participants underwent a cognitive assessment to evaluate attention, the intensity of stereotypies, the intensity of trainer aids, and global functioning before the treatment (cognitive empowerment): a total of ten of the patients were assessed with the traditional online system and ten with the ATR system. This same assessment was repeated once after treatment (post-test 1) after 5 weeks and once at 10 weeks after the conclusion of treatment (post-test 2). The scores that were obtained in the pre-test phase were compared with those that were observed in the post-test 1 assessment phase and post-test 2, to evaluate the effects of the intervention in the control group and the experimental group. To avoid subjective bias in the present study, blinded assessment was applied. One independent blinded investigator registered all the study outcomes in the three phases (Cohen’s kappa was always higher than 0.86).

### 2.3. Assessment and Measures

As stated above, all A phases of this study consisted of a cognitive assessment in which a measured the attention span, intensity of stereotypies, Global Functioning Rett Scale [31], Vineland [32], and RARS [33] of the participants in both groups. In the controls, BTR was used, in the experimental group, ATR was used.

#### 2.3.1. Attention 

The attention measurements started when the patient looked at the object of focus as requested by the educator and continued until the patient looked away from the object or stared into space. The participant was asked to look at a picture of an object (food, familiar objects, or animals) that was presented on a computer screen without eye-tracking for the control group and with eye-tracking with the experimental group. The total time that was spent by the participant looking at the stimuli or correctly at the face of the therapist was considered the parameter (in seconds).

#### 2.3.2. Type and Duration of Stereotypies

These data were recorded in a free and unstructured examination during a preliminary observation session lasting 5 min which was video recorded with a video camera. The therapists coded the data regarding the presence of stereotypies and counted the numbers of stereotypies during a 5 min period and registered the type of stereotypies.

#### 2.3.3. Global Evaluation

The Vineland Adaptive Behavior Scales-Interview second edition (VABS) and the Rett Assessment Rating Scales (RARS) were used. VABS is subdivided into four domains: communication, daily living, socialization, and motor skills. The interviewer asks general questions about the patient’s functioning in each domain and uses the responses to rate the participant on each critical behavior item (2: always present, 1: sometimes present, 0: seldom or never present). Typical interviews require approximately one hour. A total score is computed by summing the individual ratings for each scale, named the Vineland composite scores. The reliability of VABS was established as follows: split-half, 0.73–0.93 for the communication domain, 0.83–0.92 for daily living skills, 0.78–0.94 for socialization, 0.70–0.95 for motor skills, 0.84–0.98 for adaptive behavior composite, and 0.77–0.88 for maladaptive behavior (survey form) (0.80 and 0.90 for the Survey Form). The interrupter reliability coefficients for the survey and expanded forms ranged from 0.62 to 0.75. The standard error of measurement ranged from 3.4 to 8.2 over the four domains, and from 2.2 to 4.9 for the Adaptive Behavior Composite, on the survey form. 

RARS is a standardized scale that is used to evaluate patients with RTT. It is organized into seven domains: cognitive, sensorial, motor, emotional, autonomy, typical characteristics of the disease, and behavior. Typical characteristics of the disease and behavior domains measure the following characteristics: mood swings, convulsions, dyspnea, hyper-activity, anxiety, aggressivity, bruxism, oculogyric crises, epilepsy, aerophagia, muscle tension, and food preferences. A total of 31 items was generated as representative of the profile of RTT. Each item is provided with a brief glossary explaining its meaning in a few words. Each item is rated on a 4-point scale, where 1 = within normal limits, 2 = infrequent or low abnormality, 3 = frequent or medium-high abnormality, and 4 = strong abnormality. Intermediate ratings are possible; for example, an answer between 2 and 3 points is rated as 2.5. For each item, the evaluator circles the number corresponding to the best description of the patient. After a patient has been rated on all 31 items, a total score is computed by summing the individual ratings. This total score allows the evaluator to identify the level of severity of RTT, conceptualized as a continuum ranging from mild symptoms to severe deficits (Mild = 0–55; Moderate = 56–81; Severe = > 81). 

RARS was established by a standardization procedure involving a sample of 220 patients with RTT, proving that the instrument is statistically valid and reliable. More precisely, normal distribution analyses of the scores were computed and the mean scores of the scale were similar to the median and the mode. The skewness and kurtosis values, calculated for the distribution of the total score, were 0.110 and 0.352, respectively. The distribution was found to be normal. Cronbach’s alpha is used to determine the internal consistency for the whole scale and sub-scales. The total alpha was 0.912, and the internal consistency of the sub-scales is high (from 0.811 to 0.934). GAIRS [30] is a global assessment and intervention rating scales checklist for Rett syndrome coming from the items of assessment in multi-disability disorders [17,27,33,34,35,37,38,39] that was adapted for Rett syndrome. Through a global analysis, it gives an overview of the different areas and is intended for use in the functional analysis of the overall abilities of the patient. The GAIRS checklist is composed of 10 macro-areas: basic or pre-requisite behavior, neuropsychological abilities, basic cognitive concepts, advanced cognitive concepts, communication abilities, emotional- affective abilities, hand motor skills, graphomotor skills, global motor abilities, and the level of autonomy in daily life. The 10 areas are described in Table 2. 

For each area, different sequential skills, hierarchically structured, are evaluated. A total of 85 skills are evaluated. Each skill has a numerical score ranging from 1 to 5, where 1 is the minimum level of capacity and 5 is the maximum level of capacity to perform a specific activity. Below, we present some examples. In the area of basic behavior, the first skill that is evaluated is spontaneous eye contact. The score of this skill is: 1 if the child is unable to establish spontaneous eye contact, 2 if the child can establish spontaneous eye contact 2/3 times out of 10, 3 if the child can establish spontaneous eye contact 4/6 times out of 10, 4 if the child can establish spontaneous eye contact 7/8 times out of 10, and 5 if the child always establishes spontaneous eye contact. Instead, the sixth skill that is investigated in the hand motor area is grasping ability and the score is: 1 if the child cannot grasp an object on the table, 2 if the child can grasp a 5 cm object with palmar cubitus grip, 3 if the child can grasp a 5 cm object with palmar grip, 4 if the child can grasp a 1 cm object with pluri-digital grip, and 5 if the child can grasp a 1 cm object with plier’s grip (thumb-index).

#### 2.3.4. The Local Workstation

The hardware of the local workstation was composed of a laptop with an installed Skype Platform for the BTR; for the ATR, a laptop that was equipped with an eye-tracker, a webcam, a 3D camera, and a headset with a BTR system installed was used, as described below.

#### 2.3.5. Technological Architecture

**Figure 2:**
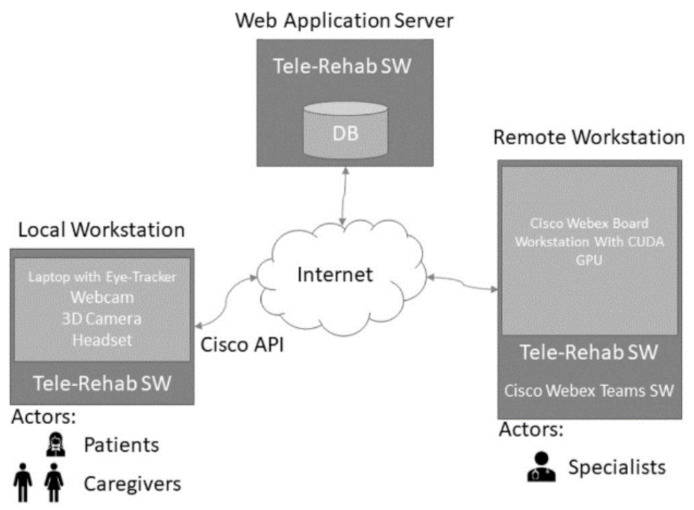
Technological architecture of the software with a web application server that was connected to both a remote workstation and a local workstation.

The ATR system (Figure 2) is composed of two types of workstations, namely a local workstation and a remote workstation.

The local workstation is intended to be used at the patient side, while the remote one is located at a specialized care center. Through the local workstation, the patient and caregiver connect with specialists for a cognitive or physical rehabilitation session. It is worth noting that, for Rett Syndrome subjects, the patient is never alone but always accompanied by a caregiver (with different skill levels, ranging from parents to therapists). The software platform is a web application, that leverages on the CISCO Webex API for network connection and video streaming. The following sections discuss the main components of the platform.

#### 2.3.6. The Telerehabilitation Software

The special requirements of patients that are affected by Rett Syndrome, and other MDs with similar characteristics, demand very specific approaches and tools. As a consequence, we designed and implemented an ad hoc architecture to manage the TR sessions and to store and analyze the acquired data that is tailored to the needs of the Rett subjects, exploiting all the knowledge that was acquired with AIRETT after years of experience in using new technologies for both cognitive and physical rehabilitation. The main characteristic of the software is that it does not interfere with the ordinary tools that the patient uses in their everyday rehabilitation activities; in addition, a caregiver can be supervised and trained by a remote specialist during the rehabilitation sessions.

The software (Associazione Italiana Rett O.N.L.U.S., Siena, Italy) is a web application with a standard set of features (such as user authentication, videoconferencing, recordings, annotations, general patients’ data) and domain-specific features. Such features exploit data acquisition and artificial intelligence techniques to add advanced tools giving therapists and caregivers a more sophisticated way to interact with the patient during the sessions and to ease further data analysis about the sessions.

More specifically, the ATR system is equipped with support for eye-tracking, so that the therapist can monitor how the patient interacts with the system during a cognitive session. In the context of motor rehabilitation, during the sessions, the movements of the patient are not the only video recorded, but there is also a 3D reconstruction that is mapped in real-time, by applying computer vision and artificial intelligence (AI) techniques. The therapist can then see the reconstructed patient’s skeleton superimposed on the patient’s image in the video stream and can even annotate if the data that are acquired during the session can be considered valid or not. The skeleton data are then used to better observe the overall patient’s pose and the angles between the bones near articulations, giving an objective measure of improvements in the patient’s movements. Moreover, we plan to apply machine learning and advanced analysis techniques to the acquired data to provide decision-making support to the specialist when the amount of data is adequate for this purpose [40].

#### 2.3.7. Procedure

This study lasted from the 1 February to mid-June 2021. The performances were recorded according to the following steps: pre-test phase; cognitive and motor empowerment; post-test phase 1 (after 5 weeks); cognitive and motor empowerment; and post test phase 2 (after 10 weeks). In the pre-test phase the AIRETT center professionals contacted the family by phone and with a brief interview that ascertained their availability for GAIRS administration sessions and all the procedures of TR. Then, the parents were invited to a session in which they completed the RARS scale that allows identifying the severity of the patients with Rett syndrome, and the Vineland questions to identify behavioral features. After these sessions, the GAIRS Checklist was administered to the patients by the AIRETT team, composed of a physician, speech therapist, and psychologist, during the evaluation sessions at the Rett Centre. All professionals had certified, special training in Rett syndrome. Some skill scores that cannot be assigned directly during the evaluation, such as the ability to go to the bathroom, were evaluated through video or interviews with parents. Every skill was requested ten times, but if the participant gave the first 3 correct answers, the skill was considered acquired; in the same way, if the participant gave the first 3 wrong answers, the skill was considered not acquired. The total administration time was around 4 hours (range from 3 to 7) but for the most seriously affected patients, it was necessary to divide checklist administration into multiple sessions (2 or 3). 

Successively, the therapist contacted the families of the control group through BTR for the GAIRS administration session and evaluation of attention and type and intensity of stereotypies, while they contacted the families of the experimental group to collect the same data through the ATR system. 

All the caregivers and the therapist received specific training in the use of BTR and ATR systems from the specialist of the AIRETT team. During all the cognitive empowerment phases, one specialist of the AIRETT Center contacted the families of the control group and the families of the experimental group to conduct a session of cognitive and motor training.

Families of both groups conducted the session three times a week with the trainer who collected data on the performance of the girls and women. Each session lasted for one hour or an hour and a half and there were 30 sessions overall that were collected. 

A session of cognitive training consisted of carrying out some cognitive discrimination tasks. The choice of the tasks depended on the level of the participant that was measured through the GAIRS Checklist. Cognitive stimuli were concrete objects or coloured pictures of objects (food, animals, toys, and familiar objects) that were presented with a distracting image in a randomized right-left order in a PowerPoint presentation. The participant was asked to look at the target, for example, an apple. Each task involved three repetitions of the presenting stimuli. The criterion allowing one to proceed to the next step was always the same: three correct answers (with eye contact) that were obtained in each of the three sessions of treatment. 

A session of motor training consisted of some global and/or fine motor exercises. Each task was chosen by the level of the participant that was measured through the GAIRS Checklist. Exercises could be a passive movement to empower the range of motion of the main articulation or motor skills, such as touch a target or walk in an open space. In motor evaluation, the score depends (a) on the number of times the participants performed the exercise correctly, for example in hand-eye coordination during motor tasks where the score went from 1, the participant never looks at her hand during the 5 motor tasks, to 5, always looks at her hands during the motor tasks; (b) on the type of performance, such as in the standing skill where the score went from 1, the participant is unable to hold the posture, to 5, holds posture without assistance for more than 3 min. During a TR session, the software allows the local caregiver to video call a remote specialist. As shown in Figure 2, once the video call starts, the software acquires video streams from the laptop screen, the laptop webcam, an external webcam, and data from the eye-tracker. The local caregiver starts a cognitive rehabilitation session with rehabilitation software that was specifically tailored for Rett patients. During the session, the patient is always in audio contact with the remote specialist, who follows what is going on through the shared screen and the videos. The specialist can also see patient interactions, i.e., the patient’s gaze on the screen. Hence, thanks to this setting and the acquired data, the specialist can monitor how the patient and the caregiver interact (from front and side views), and how the patient interacts with the rehabilitation software (through the eye-tracker pointer on screen). The parents were suggested to repeat each new learning related to motor and cognitive training in the life situations (daily training).

After the session ends, the video call is terminated and the acquired data are stored locally and shared between the caregivers and therapists through a storage cloud system. Importantly, the remote specialist can start and stop recording videos during sessions and annotate text on their own, simply by interacting with the system and without distracting the local caregiver and/or patient.

A motor TR session can be started by video calling a remote specialist, as for cognitive rehabilitation. In this case, however, there is no interaction between the patient and the local workstation. The local workstation is only used to acquire data and to let the caregiver and the specialist communicate. In the context of motor rehabilitation, the novel contribution of our approach is that during the session the movements of the patient were not only video recorded, but also reconstructed and 3D-mapped in real-time. In the post-test phase, all the participants were re-evaluated after 5 weeks and new cognitive and motor aims were established. Finally, they were re-evaluated after more than 5 weeks (post-test 2).

#### 2.3.8. Statistical Analysis

The data of each subscale of the GAIRS were obtained following standardized instructions [30] and the mean of the items for each patient was calculated for the subscales, ranging from 1 to 5 (with higher scores indicating that the patients reached the mastery performance for that subscale). The total score of GAIRS was computed by the mean of all areas.

Data analysis was performed using the IBM SPSS Statistics, Version 24 (IBM, Armonk, NY, USA). A mixed model ANCOVA for repeated measures was applied with a repeated factor time (T0–pre-intervention baseline, T1–5 weeks, post-test 1, T2–10 weeks, post-test 2), a factor group (experimental ATR, control BTR), and RARS (severity of disease at T0) as a covariate. Each of the measured parameters were the dependent variables. A Bonferroni correction was applied for multiple comparisons. The alpha level was set to *p* < 0.05 for all statistical tests. In the case of significant effects, the effect size of the test was reported. The effect sizes were computed and categorized according to eta squared η^2^ [41].

## 3. Results

The results are first discussed with reference to the level of attention and intensity of stereotypies and trainer interventions; secondly, they are analysed with reference to each subscale of GAIRS and to the total GAIRS; finally, the impact on general scales of RARS and VABS is analysed.

Table 3 summarizes means and SD (standard deviation) of attention, intensity of stereotypies, and trainer interventions at the three assessment times, and ANCOVA group-by-phase interaction results after checking for the effects of the covariate RARS. We used a mixed model ANCOVA for repeated measures with a repeated factor phase (T0–pre-intervention baseline, T1–5 weeks, post-test 1, T2–10 weeks, post-test 2), a factor group (experimental, control), and RARS as a covariate.

With reference to attention, both Phase and Group X Phase interaction showed significant effects (respectively, F (2, 46) = 3.68, *p* < 0.05, η^2^ = 0.09 and F (2, 46) = 8.91, *p* < 0.01, η^2^ = 1.11); with reference to trainer interventions, the Group X Phase interaction showed significant effects F (2, 46) = 7.48, *p* < 0.00, η^2^ = 1.03. With reference to the intensity of stereotypies (number of stereotypies that were registered in 5 min), compared with the pre-test, there were statistically significant differences in post-test 1 and post-test 2 (respectively, *p* < 0.001; *p* < 0.001); Finally with reference to trainer interventions (the number of aids or containment in 5 minutes), compared with pre-test, there were statistically significant differences in post-test 1 and post-test 2 (respectively, *p* < 0.001; *p* < 0.001). These results suggest that patients with RTT showed a positive trend in improving selective attention across time with concomitant decreases of stereotypies and trainer interventions in both groups.

With reference to GAIRS (Table 4), mixed model ANCOVAs for repeated measures with a repeated factor Phase (T0–pre-intervention baseline, T1–5 weeks, post-test 1, T2–10 weeks, post-test 2), a factor Group (experimental, control) and RARS as a covariate were applied.

Regarding the prerequisite area, the factor Phase shows a significant effect, F (2, 46) = 5.59, *p* < 0.01 η^2^ = 1.05. The interaction Group X Phase showed no significant effect, meaning that the participants of both groups significantly improved their performances in the basic behavior area, which is characterized by prerequisite behaviors for learning and communication. With reference to the neuropsychological area, the factor Phase showed a significant effect, F (2, 46) = 4.37, *p* < 0.01, η^2^ = 0.99. The interaction of Group X Phase showed no significant effect, meaning that the participants of both groups significantly improved performances in the neuropsychological area, which is characterized by brain-based skills that are needed in acquisition of knowledge, manipulation of information, and reasoning. Regarding the basic cognitive area, the factor Phase showed a significant effect, F (2, 46) = 39.04, *p* < 0.001, η^2^ = 0.88. The interaction Group X Phase showed no significant effect, meaning that the participants of both groups significantly enhanced performances in the basic cognitive area, which is characterized by the basic cognitive concepts that allow the understanding of reality (spatial concepts, topological concepts, etc.).

Regarding the advanced cognitive area, neither the factor Phase nor the interaction showed significant effects; this result may be due to the fact that not all the patients with Rett Syndrome can have access to this area as the basic cognitive area was not reached. Actually, this area evaluates the concepts of school learning that include the sub-areas of writing and mathematics. Regarding the communication area, the factor Phase showed a significant effect, F (2, 46) = 8.99, *p* < 0.001, η^2^ = 1.23. The interaction Group X Phase showed no significant effect, meaning that the participants of both groups significantly enhanced performances in the communication area, which evaluates the development of any type of language by measuring responses to environmental sounds and speech, as well as the production of sounds and words. With reference to the emotional area, the factor Phase showed a significant effect, F (2, 46) = 4.06, *p* < 0.01, η^2^ = 0.88. The interaction Group X Phase showed no significant effect, meaning that the participants of both groups significantly enhanced their performances in the emotional area, which evaluates a person’s abilities to identify emotions and express emotions. With reference to the hand motor area, the factor Phase showed a significant effect, F (2, 46) = 5.70, *p* < 0.01, η^2^ = 0.88. The interaction Group X Phase showed no significant effect, meaning again that the participants of both groups significantly enhanced performances in the hand motor area, which evaluates the ability to make movements using the small muscles in hands and wrists.

Regarding the graphomotor area, neither the factor Phase nor the interaction showed a significant effect, this may be due to the fact that not all the patients with Rett Syndrome can have access to this area as it evaluates fine motor skills incorporating graphomotor skills (GS) which, in turn, involve strength and control of finger muscles.

Regarding the global motor area, the factor Phase showed a significant effect, F (2, 46) = 4.33, *p* < 0.01, η^2^ = 0.93. The interaction Group X Phase showed no significant effect, meaning that the participants of both groups significantly enhanced their performances in the global motor area, which evaluates gross-motor skills which are important for an upright posture, walking and running.

Regarding autonomy in daily life area, neither the factor Phase nor the interaction shows a significant effect, for some patients with Rett Syndrome it is too hard to reach adaptive and self-help behavior at home, as well as social behavior that develops through early adult-child interactions and therefore the level of autonomy in the praxis of daily life.

With reference to the total score of GAIRS, the factor Phase showed a significant effect, F (2, 46) = 6.87, *p* < 0.01, η^2^ = 0.88. The interaction Group X Phase also showed a significant effect F (2, 46) = 9.87, *p* <.001, η^2^ = 1.11, meaning that the participants of the experimental group significantly enhanced their performances in all areas more than the control group.

With reference to VABS and RARS total scores, Table 5 shows that the scores at the pre-test were significantly different compared with those at post-test 1 and post-test 2 (*p* < 0.001). Only the Phase factor showed significant effects (VABS, F (2, 46) = 9.87, *p* < 0.01, η^2^ = 1.01; RARS, F (2, 46) = 5.99, *p* < 0.01, η^2^ = 0.94). These results indicate an increase in the global functioning in the three phases of treatment and a general decrease in the level of severity of RTT.

## 4. Discussion

Telerehabilitation (TR) delivers rehabilitation and therapeutic services with the support of diverse technologies. Concerning the field of neurodevelopmental disorders (NDDs), a recent systematic review has demonstrated that TR is an effective tool in improving the adaptive skills of children with NDDs (Woolf et al., 2016). 

Although the use of TR for NDDs has grown rapidly, few studies use TR intervention for children with Rett Syndrome (RTT) [20,25,26,28,42,43,44,45]. The main aim of the present study was to determine whether the use of TR with advanced technology (ATR) in patients with RTT leads to greater improvements in motor and cognitive functions than with the use of basic TR (BTR). 

Both the ATR and BTR groups increased time of attention while the educational interventions and intensity of stereotypies decreased. Regarding the results of the GAIRS assessment, again it was shown that both the ATR and BTR groups increased their performances. Since both groups equally benefitted from ATR and BTR, our hypothesis is not confirmed. The advanced system (with eye-tracking and 3D-mapped in real-time) did not empower the performances of the girls and women with RTT any better than the basic system. We think that the explanation may be related to the complexity of RTT; the treatments for these girls and women require a multidisciplinary team, with specialists coming from different areas - medical, psychological, social, educational, and occupational - and are directed toward symptoms and providing support in different areas of daily life. One of the most important factors in cognitive and motor rehabilitation is the high intensity and frequency of treatment; to reach functional educational goals, frequency is a critical factor [28,46] in both the ATR and BTR groups the training frequency was three times a week. The parents were invited also to follow the training with practical exercises as described in the procedure section.

Unlike other studies that were carried out in past years, the results of rehabilitation improvement were also demonstrated by clinical evaluation using RARS and Vineland Scales. The RARS scale showed a reduction in score that was more marked for the ATR group, which is equivalent to a reduction in the severity of the syndrome and the Vineland scale emphasizes an improvement in the overall functioning of the participants at the end of the training. These results show a generalization of skills that are learned outside the purely rehabilitative field, which is demonstrated by GAIRS [30].

The results show that the improvements have been progressive and continuous throughout the project, as demonstrated by the results in post-test 1 and post-test 2, for the GAIRS, RARS, and Vineland scores, where the improvement of the parameters was more marked in post-test 2.

These results should be interpreted in the light of a number of limitations. The present study involved 20 subjects, which is not a small group for RTT, given that RTT is a rare disorder; but caution is required in the interpretation of results for potential problems in generalization. In addition, we didn’t use a waiting group; it might be worthwhile having a waiting group who are ready and who, therefore, would not require training. Given that learning times in cognitive and motor disabilities are longer compared to typically developing subjects, it is important to set up a longer training period and include follow-up phases to show maintenance of the results in the medium- and long-term.

## 5. Conclusions

The main aim of the present study was to determine whether the use of TR with advanced technology (ATR) in patients with RTT leads to greater (or at least equal) improvements in motor and cognitive functions than basic TR (BTR).

The results indicated that telerehabilitation is an effective mode of rehabilitation for Rett syndrome as it allows high intensity and high frequency interventions, which has proven to be the most critical factor. The results did not reveal a substantial difference between the two groups with regard to the overall rehabilitation prospects that were assessed through GAIRS and the overall functioning of the participants that was assessed through RARS and Vineland; however, a more marked increase was seen for the ATR group compared to the BTR group for behavioral parameters with an increase in sustained attention to the task and a reduction in educational interventions and stereotypies. The results demonstrate an important aspect of generalizing the skills that are learnt from training, as there have also been increases in RARS and Vineland scores.

As stated in the introduction, few studies have used TR in patients with RTT and no study has compared the effectiveness of basic and advanced TR in this population, so future research is necessary to better understand the characteristics of effective TR interventions in these patients and to determine how these characteristics may differ for specific populations and outcomes. Future studies should involve a larger number of participants, also those with other MECP2 genetic mutations. To advance the field, we encourage authors of future research to use ATR to determine best practices and to render this type of TR more effective and cost-effective.

## Figures and Tables

**Table 1:** Characteristics of participants.

Participants	Name	Clinical Stage	Age	MeCP2 Mutation	Level of Severity (RARS)	Functional Ability Level
ATR Group						
1	L.G	IV	25	T158M	75.5	75
2	L.A	IV	25	T158M	75.5	75
3	D.D	IV	31	R306C	75	90
4	C.A	III	5	T158M	58	84
5	A.C	III	5	----	71	71
6	C.L	III	4	P152R	69.5	109
7	F.D	IV	18	T158M	64	136
8	S.M	III	14	T158M	62	91
9	D.F	IV	25	R255X	64	111
10	C.M	III	7	P322L	65.5	104
11	S.D	IV	15	P133C	72	151
BTR Group						
1	B.C	III	5	R255X	71	75
2	S.A	III	10	P322L	75	108
3	B.G	IV	24	P152R	75	74
4	G.L	IV	10	R255X	75.5	84
5	S.L	IV	9	T158M	70	78
6	B.A	III	10	P152R	75	71
7	P.V	III	8	----	65.5	69
8	L.M	III	9	P322L	58	136
9	M.S	IV	24	T158M	64	110
10	S.P	IV	22	T158M	62	105

**Table 2:** Description of GAIRS checklist areas.

1. Basic Behaviors Area: Evaluates the prerequisite behaviors for learning and communication, they are: spontaneous eye contact, eye contact on request, looking at objects, tracking objects and faces, functional gestures, cooperation with simple spoken requests (reply to their name, look for mother), sitting long enough to complete a task, object permanence, be able to wait for their turn before starting an activity, and be able to communicate basic needs (need to eat, drink, sleep, play, walk, go to the bathroom, and feel good or bad).
2. Neuropsychological Area: Evaluates brain-based skills which are needed in acquisition of knowledge, manipulation of information, and reasoning. They have more to do with the mechanisms of how people learn, remember, problem-solve, and pay attention, rather than with actual knowledge. This area includes selective attention, types and intensity of stereotypes, lateralization, temporal orientation, spatial orientation, memory span, logical sequences, and categorization (animals, dress, foods, drinks, objects, places, actions).
3. Basic Cognitive Area: Evaluates the basic cognitive concepts that allow the understanding of reality (spatial concepts, topological concepts, etc.). This area includes object recognition, color discrimination, geometric form discrimination measure concepts, spatial concepts, human body discriminations, time concepts, and cause-effect relationship.
4. Advanced Cognitive Area: Evaluates the concepts of school learning that include the sub-areas of writing and mathematics. This area includes global words recognition, syllables recognition, recompleting words through syllables, alphabetic symbols recognition, recompleting words with alphabetic symbols, recognition of words representing actions, using words to communicate, math pre-requisite concepts, recognition of numbers, and biunivocal relation between number and quantity.
5. Communication Area: Evaluates the development of language by measuring responses to environmental sounds and speech, as well as the production of sounds and words. The skills of communication, comprehension and expression that allow the person to interact with others. This area includes expressing a basic need at a corporal level, recognizing, and expressing a basic need through pictures, understanding the biunivocal relation between the corpora, recognizing and expressing a basic need through word, understanding the biunivocal relation of a basic need between a picture and the word that expresses it, verbal comprehension, and verbal production.
6. Emotional Area: Evaluates the person’s abilities and ways of experiencing, expressing, and understanding their own emotions and those of others are analyzed. This area includes identify emotions and express emotions.
7. Hand motor Area: Evaluates the ability to make movements using the small muscles in our hands and wrists. Children rely on these skills to do key tasks in school and in everyday life. Fine motor skills are complex, however. They involve the coordinated efforts of the brain and muscles and they are built on the gross motor skills that allow us to make bigger movements. This area includes musculoskeletal alterations, hand-eye coordination during motor tasks lateralization, reaching movement, touching ability, grasping ability, releasing movement, repositioning movement, bimanual coordination, and the ability to push and pull an object.
8. Graphomotor Area: Evaluates the fine motor skills incorporating, among others, graphomotor skills (GS) which, in turn, involve strength and control of the finger muscles, and incorporates important daily skills such as writing and drawing that are necessary for the academic achievement of all students. This area includes grasping of pencil, drawing patterns, and the use of school tools.
9. Global Motor Area: Evaluates the gross-motor skills which are important for an upright posture, walking, running, and climbing. It allows for the observation of physical weakness or disability or defects of movement. This area includes: standing, sitting, parachute reactions, rolling supine—on one side, rolling supine—prone, supine—to seated on the floor, seated on the floor—to standing, seated on a chair—to standing, standing—to seated on the floor, standing—to seated on a chair, walking, body spatial orientation in standing, stepping, running, climbing upstairs, descending stairs, jumping, picking up an object from the ground (small ball), playing with a ball, and walking on a slope.
10. Autonomy in Daily Life Area: Measures early adaptive and self-help behavior typically seen at home, as well as social behavior that develops through early adult-child interactions; therefore, this area analyses the level of autonomy in the praxis of daily life This area includes daily autonomy such as, eating, drinking, coughing or difficulty breathing during meal, type of food’s consistence, washing, autonomy in the bathroom and dressing, and other skills such as, playing and socialization skills, and advanced autonomy activities.

**Table 3:** The means and standard deviations of attention, intensity of stereotypies and trainers’ interventions.

	Pre-Test		Post-Test1		Post-Test2		*p*
Measures	Experimental	Control	Experimental	Control	Experimental	Control	
Attention time	11.36 (7.88)	12.17 (7.33)	18.82 (8.74)	16.67 (7.37)	29.64 (8.64)	19.17 (4.92)	0.05
Intensity of trainer aids	21.91 (4.46)	22.50 (7.58)	10.09 (3.86)	18.83 (9.40)	8.64 (4.93)	16.83 (7.78)	0.00
Intensity of stereotypes	2.64 (1.12)	2.87 (1.11)	2.91 (1.04)	2.43 (0.98)	3.00 (1.00)	2.86 (1.07)	0.001

**Table 4:** The means and standard deviations of GAIRS’ Areas.

	Pre-Test		Post-Test1		Post-Test2		*p*
Measures	Experimental	Control	Experimental	Control	Experimental	Control	
Basic Behaviors Prerequisites Area	3.58 (0.47)	3.47 (0.59)	4.06 (0.42)	3.72 (0.53)	4.24 (0.27)	3.92 (0.41)	0.01
Neuropsychological Area	1.90 (0.28)	1.72 (0.48)	2.45(0.40)	2.05(0.55)	2.86 (0.50)	2.29 (0.66)	0.01
Basic Cognitive Area	2.29 (0.84)	2.06 (0.92)	2.79 (0.74)	2.45 (0.88)	3.22 (0.74)	2.79 (0.88)	0.001
Advanced Cognitive Area	1.00 (0.00)	1.08 (0.18)	1.01 (0.03)	1.10 (0.17)	1.03 (0.07)	1.15 (0.17)	0.08
Communication Area	2.16 (0.44)	1.87 (0.59)	2.60 (0.37)	2.23 (0.69)	2.81 (0.35)	2.37 (0.79)	0.001
Emotional Area	2.88 (0.58)	2.92 (0.74)	3.38 (0.74)	3.25 (0.82)	3.66 (0.76)	3.42 (0.75)	0.01
Hand motor Area	2.82 (0.67)	2.87 (0.56)	3.27 (0.83)	3.20 (0.52)	3.52 (0.78)	3.41 (0.61)	0.01
Graphomotor Area	1.36 (0.41)	1.24 (0.37)	1.55 (0.56)	1.38 (0.36)	1.58 (0.56)	1.48 (0.57)	0.09
Global Motor Area	2.61 (0.32)	3.01 (0.40)	2.85 (0.32)	3.19 (0.30)	2.94 (0.32)	3.26 (0.29)	0.01
Autonomy in Daily Life Area	2.37 (0.41)	2.10 (0.70)	2.42 (0.38)	2.15 (0.74)	2.47 (0.39)	2.17 (0.77)	0.08
Total Score GAIRS	2.29 (0.23)	2.30 (0.44)	2.66 (0.27)	2.51 (0.44)	2.84 (0.26)	2.67 (0.49)	0.01

**Table 5:** Means and standard deviations of Vineland and RARS total score.

	Pre-Test		Post-Test1		Post-Test2		*p*
Measures	Experimental	Control	Experimental	Control	Experimental	Control	
Vineland Score	98.70 (26.95)	95.25 (4.57)	102.50 (25.23)	97.25 (7.50)	105.60 (26.30)	95.24 (6.44)	0.001
RARS Score	67.70 (5.90)	67.00 (8.80)	65.75 (6.58)	66.30 (9.10)	64.60(5.80)	65.90 (9.70)	0.01

## Data Availability

Data available on request due to restrictions e.g., privacy or ethical.
